# Downregulation of leaf flavin content induces early flowering and photoperiod gene expression in Arabidopsis

**DOI:** 10.1186/s12870-014-0237-z

**Published:** 2014-09-09

**Authors:** Hongtao Ji, Yueyue Zhu, Shan Tian, Manyu Xu, Yimin Tian, Liang Li, Huan Wang, Li Hu, Yu Ji, Jun Ge, Weigang Wen, Hansong Dong

**Affiliations:** Plant Growth and Defense Signaling Laboratory, State Ministry of Education Key Laboratory of Integrated Management of Crop Pathogens and Insect Pests, Nanjing Agricultural University, Nanjing, 210095 China

## Abstract

**Background:**

Riboflavin is the precursor of flavin mononucleotide (FMN) and flavin adenine dinucleotide (FAD), essential cofactors for many metabolic enzymes that catalyze a variety of biochemical reactions. Previously we showed that free flavin (riboflavin, FMN, and FAD) concentrations were decreased in leaves of transgenic Arabidopsis plants expressing a turtle riboflavin-binding protein (RfBP). Here, we report that flavin downregulation by RfBP induces the early flowering phenotype and enhances expression of floral promoting photoperiod genes.

**Results:**

Early flowering was a serendipitous phenomenon and was prudently characterized as a constant phenotype of RfBP-expressing transgenic Arabidopsis plants in both long days and short days. The phenotype was eliminated when leaf free flavins were brought back to the steady-state levels either by the *RfBP* gene silencing and consequently nullified production of the RfBP protein, or by external riboflavin feeding treatment. RfBP-induced early flowering was correlated with enhanced expression of floral promoting photoperiod genes and the florigen gene *FT* in leaves but not related to genes assigned to vernalization, autonomous, and gibberellin pathways, which provide flowering regulation mechanisms alternative to the photoperiod. RfBP-induced early flowering was further correlated with increased expression of the *FD* gene encoding bZIP transcription factor FD essential for flowering time control and the floral meristem identity gene *AP1* in the shoot apex. By contrast, the expression of *FT* and photoperiod genes in leaves and the expression of *FD* and *AP1* in the shoot apex were no longer enhanced when the *RfBP* gene was silenced, RfBP protein production canceled, and flavin concentrations were elevated to the steady-state levels inside plant leaves.

**Conclusions:**

Token together, our results provide circumstantial evidence that downregulation of leaf flavin content by RfBP induces early flowering and coincident enhancements of genes that promote flowering through the photoperiod pathway.

**Electronic supplementary material:**

The online version of this article (doi:10.1186/s12870-014-0237-z) contains supplementary material, which is available to authorized users.

## Background

Riboflavin (vitamin B2) is the precursor of flavin mononucleotide (FMN) and flavin adenine dinucleotide (FAD), essential cofactors for many metabolic enzymes implicated in multiple cellular processes [1–3]. Plants can synthesize riboflavin while the levels vary widely in different organs and during different stages of development, suggesting that changes in riboflavin levels may cause physiological effects [2,4,5]. Foliar application of riboflavin increases the intrinsic concentrations of all flavins (riboflavin, FMN, and FAD), alters cellular redox, and induces defense responses to pathogens [6–10]. The foliar flavin content can be also modulated by transgenic expression of the turtle (*Trionyx sinensis japonicus*) gene encoding riboflavin-binding protein (RfBP) [11]. The protein contains a nitroxyl-terminal (N-terminal) ligand-binding domain, which is implicated in molecular interactions, and a carboxyl-terminal (C-terminal) phosphorylated domain, which accommodates the riboflavin molecule [12–15]. In the *RfBP*-expressing (RfBP^+^) *Arabidopsis thaliana* line, the RfBP protein localizes to chloroplasts, binds with riboflavin to decrease free flavin concentrations in leaves, and enhances the plant resistance to diseases [11]. The induction of disease resistance accompanies elevated cytosolic levels of hydrogen peroxide (H_2_O_2_), a cellular signal that can regulate defense responses [7,10,11,16]. All of these RfBP-conferred responses can be eliminated by nullifying *RfBP* expression and abolishing production of the RfBP protein. The *RfBP*-silenced (RfBP^−^) Arabidopsis line generated under RfBP^+^ background resembles the wild-type (WT) plant in the leaf flavin content, disease resistance, and H_2_O_2_ production [11]. These findings support the notion that changing flavin concentrations has biological consequences [7,10,11].

RfBP is a phosphoglycoprotein that was first isolated from the white of chicken egg [17] and then identified in different species of both ovipara and mammals, such as emu [18], amphibian [19], fish [20], and humans [21]. In ovipara, the *RfBP* gene is expressed in the liver and oviduct in an estrogen-dependent manner, and is also expressed in oocytes subsequent to fecundation [12,18,22]. The estrogen-dependent and fecundation-induced expression patterns are also found in mammals [21]. Regarding to the RfBP protein, it is mainly produced in the blood plasma of podocyte and localizes to the plasma membrane via the N-terminal ligand-binding domain [23,24]. RfBP also employs the C-terminal phosphorylated domain to tightly bind riboflavin in a 1:1 molar ratio [24–26]. Owing to these features, RfBP functions to mediate the cellular translocation of riboflavin in the animals [27,28]. The animals absorb riboflavin directly from dietary sources [29] or produce this vitamin through conversions from ingested FMN and FAD [1,30]. In both cases, RfBP acts to redistribute riboflavin between cells and organs [13,27]. Moreover, RfBP adopts a ligand-receptor binding manner [13,31,32] to mediate riboflavin translocation into the growing embryo [25]. Either riboflavin deficit or insufficient decomposition of the riboflavin-RfBP complex is fatal to embryogenesis [33]. These findings suggest that RfBP plays an important role in the animal development. In agreement with this role, we unexpectedly found that the Arabidopsis RfBP^+^ line flowered earlier than WT and RfBP^−^ plants [11]. This serendipitous phenomenon suggests that the de novo expression of RfBP may affect the regulation of flowering time in the plant.

Plant flowering time is mainly controlled by four genetic pathways that are well characterized in Arabidopsis [34,35]. The photoperiod and vernalization pathways regulate flowering in response to the length of the day and a long period of cold, respectively [36,37]. The gibberellin (GA) pathway refers to the requirement of GA for normal flowering patterns [35,36]. The autonomous pathway indicates flowering regulation in a photoperiod and GA independent manner [37]. These pathways may interact [34,35] through multiple regulators, such as the putative zinc finger transcription factor CO (CONSTANS) [38], the florigen protein FT (FLOWERING LOCUS T) [39], and the circadian clock oscillators TOC1 (TIMING OF CAB EXPRESSION1) and CCA1 (CIRCADIAN CLOCK-ASSOCIATED1) [40]. As a result, the expression of floral meristem identity (FMI) genes, such as *AP1* (*APETALA1*) [41], is induced at the shoot apex to promote the growth of floral organ primordia, which form flowers in the subsequent days [42,43]. A main purpose of this study was to elucidate which of the four floral pathways is related to the early flowering phenotype associated with downregulation of free flavin concentrations by RfBP.

## Results

### RfBP reduces leaf flavin content in long days and short days

Recently we showed that leaf flavin (riboflavin, FMN, and FAD) concentrations were significantly reduced in the Arabidopsis RfBP^+^ (synonym REAT11) line than in WT or RfBP^−^ (synonym RfBPi11) plants under a 12-hour light/12-hour dark cycle [11]. This photoperiod is not well suited for the study of flowering regulation, but instead, short day is specified to be an 8-hour light/16-hour dark cycle while long day indicates 16-hour light [34]. Therefore, we changed to grow WT, RfBP^+^, and RfBP^−^ plants under long day (16-hour light) and short day (8-hour) conditions, respectively. We retested the *RfBP* gene expression, RfBP protein production, and free flavin concentrations in the two youngest expanded leaves of 10-day-old plants from long days and 25-day-old plants from short days according to flowering time of the different plants (see below).

**Figure 1:**
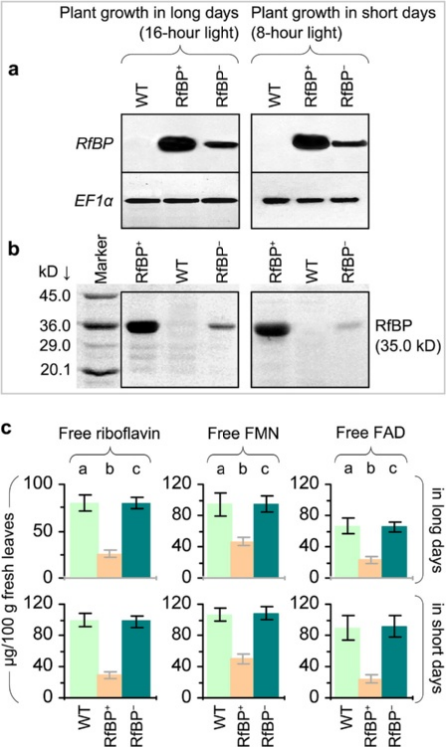
***RfBP*** **expression and flavin content in leaves of the wild-type (WT) plant and** ***RfBP*** **-expressing (RfBP** ^**+**^ **) or** ***RfBP*** **-silencing (RfBP** ^**−**^ **) line of Arabidopsis.** Plants were grown for 10 days in long days (16 hour light) or 25 days in short days (8 hour) before use in the following analyses. **(a)** Northern blotting with the probe specific to the *RfBP* gene or the constitutively expressed *EF1α* gene used as a reference. **(b)** Analysis of plant proteins by the gel electrophoresis. Protein bands were visualized by gel staining with Coomassie G-250. Molecular makers are indicated. **(c)** Quantification of flavin concentrations. Data shown are mean values ± standard deviation bars of results from three independent experiments each containing three repeats and 15 plants per repeat. Different letter on bar graphs indicate significant differences by analysis of variance and least significant difference test (*P* < 0.01).

In parallel tests of plants under long days or short days, the *RfBP* gene was highly expressed (Figure 1a) and a substantial amount of the RfBP protein was produced (Figure 1b) in leaves of RfBP^+^ in contrast to the absence of gene expression and protein production in the WT plant. The gene expression and protein production were highly reduced in the RfBP^−^ plant (Figure 1a,b). In long days, free riboflavin, FMN, and FAD concentrations were decreased by 60%, 52%, and 69%, respectively, in leaves of RfBP^+^ compared to WT, but in RfBP^−^, flavins were retrieved to approximations of WT levels (Figure 1c). Similar differences were found in *RfBP* expression (Figure 1a), the protein production (Figure 1b), and flavin concentrations (Figure 1c) among WT, RfBP^+^, and RfBP^−^ under short days. Leaf flavin concentrations were decreased approximately by 20% in all plants grown in long days compared to short days (Figure 1c). These analyses suggest that downregulation of free flavin concentrations in leaves is a constant character of the RfBP^+^ plant under short day and long day conditions.

### Downregulation of leaf flavin content causes early flowering

**Figure 2:**
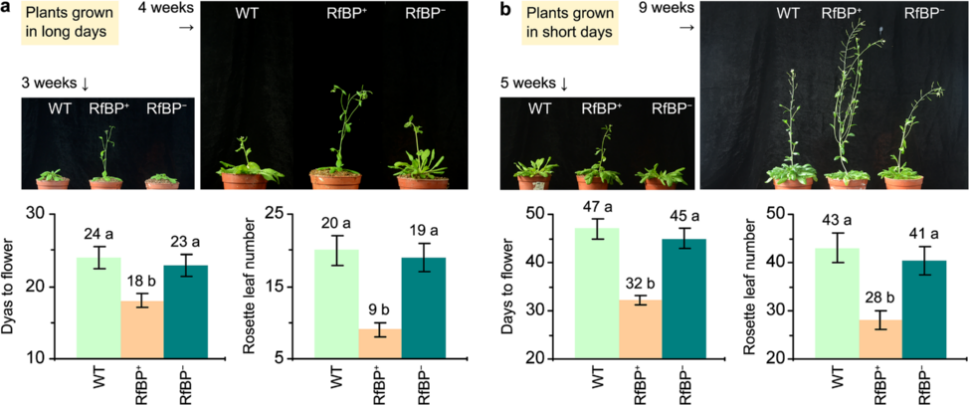
**Flowering characters of WT, RfBP** ^**+**^ **, and RfBP** ^**−**^ **plants.** Plants were grown in long days **(a)** and short days **(b)**, respectively. Data shown in bar graphs are mean values ± standard deviation bars of results from three independent experiments each containing three repeats and 30 plants per repeat. Observed values are shown on deviation bars. Different letters in bar graphs indicate significant differences by analysis of variance and least significant difference test (*P* < 0.01).

The WT plant took 24 and 47 days to flower with 20 and 43 rosette leaves in long days (Figure 2a) and short days (Figure 2b), respectively. RfBP^−^ resembled WT in flowering time and rosette leaf number but RfBP^+^ flowered 6 days earlier with a reduction of 11 rosette leaves in long days (Figure 2a) and flowered 15 days earlier with a shortage of 15 rosette leaves in short days (Figure 2b). Like RfBP^+^, other *RfBP*-expressing lines [11] also acquired the early flowering phenotype (Additional file 1: Figure S1). Thus, early flowering is a constant character of *RfBP*-expressing plants.

**Figure 3:**
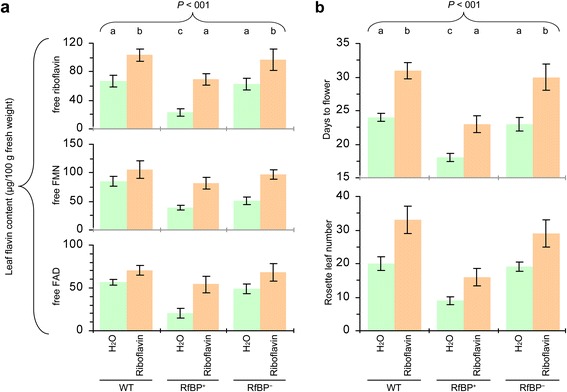
**The effects of riboflavin feeding on leaf flavin content and plant flowering time under long days.** Ten-day-old plants grown in long days were fed with riboflavin or treated with water in control. Two days later, leaf flavin content was determined **(a)**. Subsequently, plant flowering time and rosette leaf number were scored **(b)**. Data shown in bar graphs are mean values ± standard deviation bars of results from three independent experiments each containing three repeats and 15 plants per repeat. Different letters on top indicate significant differences by analysis of variance and least significant difference test.

To elucidate the effect of leaf flavin concentrations on flowering, we performed a pharmacological study in which plants under long days were fed with an aqueous solution of riboflavin or treated with ultrapure water as a control. Riboflavin feeding caused substantial increases in the leaf content of all flavins, and flavin concentrations in riboflavin-fed RfBP^+^ were retrieved to the approximations in water-treated WT plants (Figure 3a). RfBP^−^ resembled WT in the riboflavin-feeding effects on leaf flavin content (Figure 3a). All plants flowered later and had more rosette leaves following riboflavin feeding compared to control while riboflavin-fed RfBP^+^ plants lost the early flowering phenotype (Figure 3b). These observations are in agreement with the *RfBP* silencing effect and both lines of evidence attribute the early flowering phenotype to the reduction of leaf flavin concentrations.

### Flavin content downregulation enhances foliar expression of floral promoting photoperiod genes

To infer the molecular basis of RfBP-induced early flowering, we compared WT, RfBP^+^, and RfBP^−^ plants in terms of the expression of 14 flowering regulatory genes assigned to photoperiod (*PHYA*, *PHYB*, *CRY1*, *CRY2*, *CCA1*, *TOC1*, and *CO*), vernalization (*FLC*, *FRI*, and *VIN3*), GA (*GA1* and *GAI*), and autonomous (*FLC* shared with vernalization, *FLM*, and *LD*) pathways. Plants were grown in long days and sampled during 10–30 days after seed germination. Gene expression was analyzed by quantitative real-time reverse transcriptase-polymerase chain reaction (RT-PCR) using constitutively expressed *EF1α* and *Actin2* genes as references. RNAs used in the analysis were isolated from the two youngest expanded leaves at 13 hours in light (three hours to dark), a time point at which floral promoting genes are highly expressed under regulation of the circadian clock, a central player in the photoperiod pathway [34,35].

**Figure 4:**
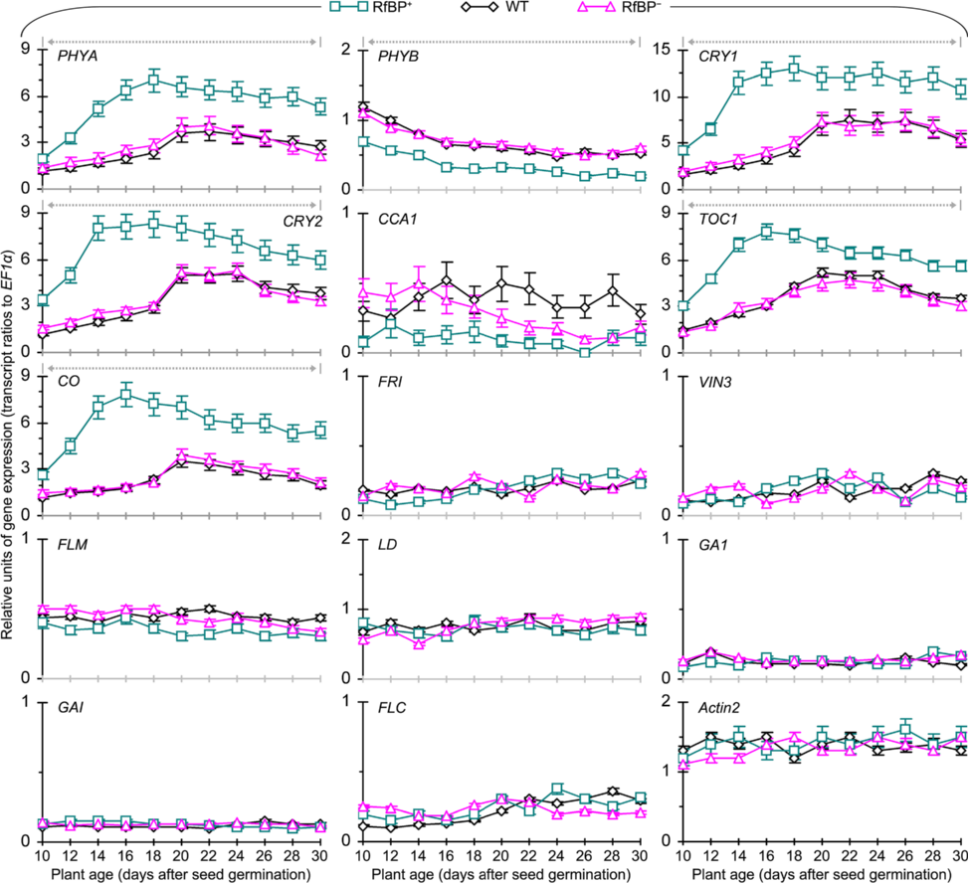
**The expression of flowering regulatory genes in leaves of WT, RfBP** ^**+**^ **, and RfBP** ^**−**^ **plants grown in long days.** Chronological patterns of gene expression were analyzed by quantitative real-time RT-PCR with RNAs isolated from the two youngest leaves of plants at the indicated times. The constitutively expressed *EF1α* and *Actin2* genes were used as references. Data shown in curves are mean values ± standard bars of results from three independent experiments each containing three repeats and five plants per repeat. Gray dashed and bidirectional arrowheads indicate significant differences between RfBP^+^ and WT or RfBP^−^ at the range of time intervals based on analysis of variance and least significant difference test (*P* < 0.01).

Chronological patterns of gene expression analyzed every other day during 10–30 days of plant growth are provided in Figure 4. The seven genes assigned to the vernalization, GA, or autonomous pathway were little expressed in all plants while the seven photoperiod genes behaved differently. Regarding to photoperiod, red/far red light receptor phytochromes PHYA and PHYB [44,45] and blue light receptor cryptochromes CRY1 and CRY2 [46,47] serve as the entry of the clock [40], which employs the negative *CCA1* and *TOC1* transcriptional feedback loop to control day-night rhythm of photoperiod gene expression [40,48,49]. RfBP did not cause evident effect on *CCA1* as its expression levels were similar in all plants through out the course of time. *PHYB* expression was decreased with time in all plants but decreasing extents were significantly (*P* < 0.01) smaller in RfBP^+^ compared to WT or RfBP^−^. Expression levels of five other photoperiod genes (*PHYA*, *CRY1*, *CRY2*, *TOC1*, and *CO*), which are flowering activators [34,38,40,44,48,50], were highly elevated as compared to controls (*Actin2* to *EF1α* transcript ratios) and sharp elevations were detected approximately four days before flowering in all plants. However, RfBP^+^ was more vigorous than WT and RfBP^−^ in chronologically increased expression of the photoperiod genes. Their expression was highly enhanced in RfBP^+^ compared to WT or RfBP^−^ at every time point during 10–30 days. During this period multiples of expression enhancements by RfBP were 1.4–3.2 for *PHYA*, 1.6–4.4 for *CRY1*, 1.4–4.0 for *CRY2*, 1.5–2.8 for *TOC1*, and 1.9–4.5 for *CO*. Clearly, RfBP^+^ enhances the foliar expression of floral promoting photoperiod genes (Figure 4).

The effect of RfBP on gene expression was cancelled by the riboflavin feeding treatment (Additional file 2: Figure S2), which annulled the early flowering phenotype and also eliminated approximate RfBP-reduced parts of the intrinsic flavin content in RfBP^+^ leaves (Figure 3b). The endogenous flavin concentrations were increased (Figure 3a) and expression levels of the five floral promoting photoperiod genes were decreased significantly (*P* < 0.01) in leaves of WT and RfBP^−^ plants fed with riboflavin compared to water (Additional file 2: Figure S2). Therefore, free flavin concentrations negatively affect RfBP-enhanced expression of the floral promoting photoperiod genes in leaves with long days.

### RfBP enhances *FT* expression in leaves and coordinate *FD* and *AP1* expression in the shoot apex

**Figure 5:**
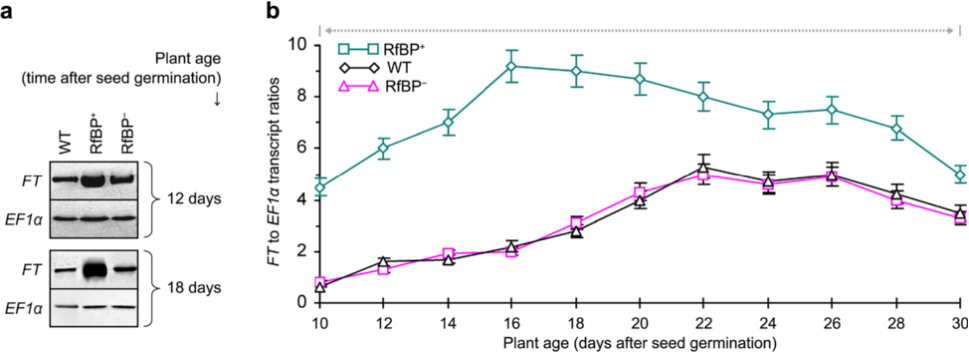
**Expression of the florigen gene** ***FT*** **in leaves of the different plants grown in long days.** Gene expression was analyzed by Northern blotting **(a)** and quantitative real-time RT-PCR (b). Both analyses were performed on RNAs isolated from the two youngest leaves of plants at the indicated times and using *EF1α* and *Actin2* genes as references. Data shown in curves **(b)** are mean values ± standard bars of results from three independent experiments each containing three repeats and five plants per repeat. Gray dashed and bidirectional arrowheads indicate significant differences between RfBP^+^ and WT or RfBP^−^ at the range of time intervals based on analysis of variance and least significant difference test (*P* < 0.01).

The circadian clock exit gene *CO* [48] is one of RfBP-induced photoperiod genes (Figure 4). In response to the photoperiod signal, CO is produced as an output of the circadian clock and acts in turn to activate the expression of the florigen gene *FT* in leaves [48,50]. As shown in Figure 5a, marked expression of *FT* was detected in leaves of 12- and 18-day-old plants with greater quantities in RfBP^+^ than in WT or RfBP^−^ under long day condition. Interestingly, *FT* still displayed substantial expression in RfBP^+^ on the flowering day (Figure 5a compared to Figure 2a). As shown in Figure 5b, quantities of the *FT* transcript in the different plants with long days were markedly increasing since 10 days of growth, reached the highest values on two days before flowering, and started to decline gradually after flowering. Thus, chronological patterns of *FT* expression were similar in all plants during 10–30 days of growth in long days. However, *FT* expression levels kept greater at every time point and was increased earlier with significantly (*P* < 0.01) higher extents in leaves of RfBP^+^ compared to WT and RfBP^−^ (Figure 5b).

**Figure 6:**
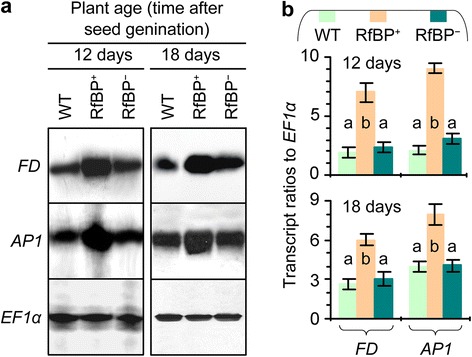
**Expression of floral meristem identity genes** ***FD*** **and** ***AP1*** **in shoot apices of plants grown in long days.** Northern blotting **(a)** and real-time RT-PCR **(b)** analyses were performed with RNAs isolated from shoot apices of plants at the indicated times. Data shown in **(b)** are mean values ± standard deviation bars of results from three independent experiments each with three repeats and five plants per repeat. Different letters in bar graphs indicate significant differences by analysis of variance and least significant difference test (*P* < 0.01).

As a result of the photoperiod regulation, the florigen FT protein moves from leaves to the shoot apex [51,52], where it functions with FD to activate AP1 [12,13], which marks the beginning of floral organ formation [34]. At the transcription level, the *FD* and *AP1* genes are coordinately expressed at the shoot apex to initiate flowering by promoting the growth of floral organ primordia [42,43]. To elucidate the role of *FD* and *AP1* in RfBP-induced flowering, we analyzed their expression in shoot apices of 12- and 18-day-old plants. We detected concomitant expression of *FD* and *AP1* from all plants (Figure 6a) and significantly (P *<* 0.01) higher amounts of gene transcripts in RfBP^+^ than in WT or RfBP^−^ (Figure 6b). Clearly, the de novo expression of RfBP affects the synchronized expression of *FD* and *AP1* at the shoot apex.

### RfBP enhances expression of photoperiod and *FT* genes in leaves and expression of *FD* and *AP1* in shoot apices under inductive photoperiod

To further elucidate the molecular basis of RfBP-induced early flowering, we tested the expression of *FT* and flowering regulatory genes in leaves and the expression of *FD* and *AP1* in shoot apices of WT, RfBP^+^, and RfBP^−^ plants under inductive photoperiod. This condition was devised by considering: (i) RfBP^+^ flowers after 32 days while WT and RfBP^−^ flower after 47 and 46 days of growth in short days (Figure 2b); and (ii) floral organ primordia can well grow within five days and differentiate into floral organs in the subsequent days under inductive photoperiod [43]. Therefore, we employed the inductive photoperiod by growing plants in short days for 23 days and transferred them to long days. We analyzed gene expression immediately (zero day) after inductive photoperiod and in the subsequent nine days.

**Figure 7:**
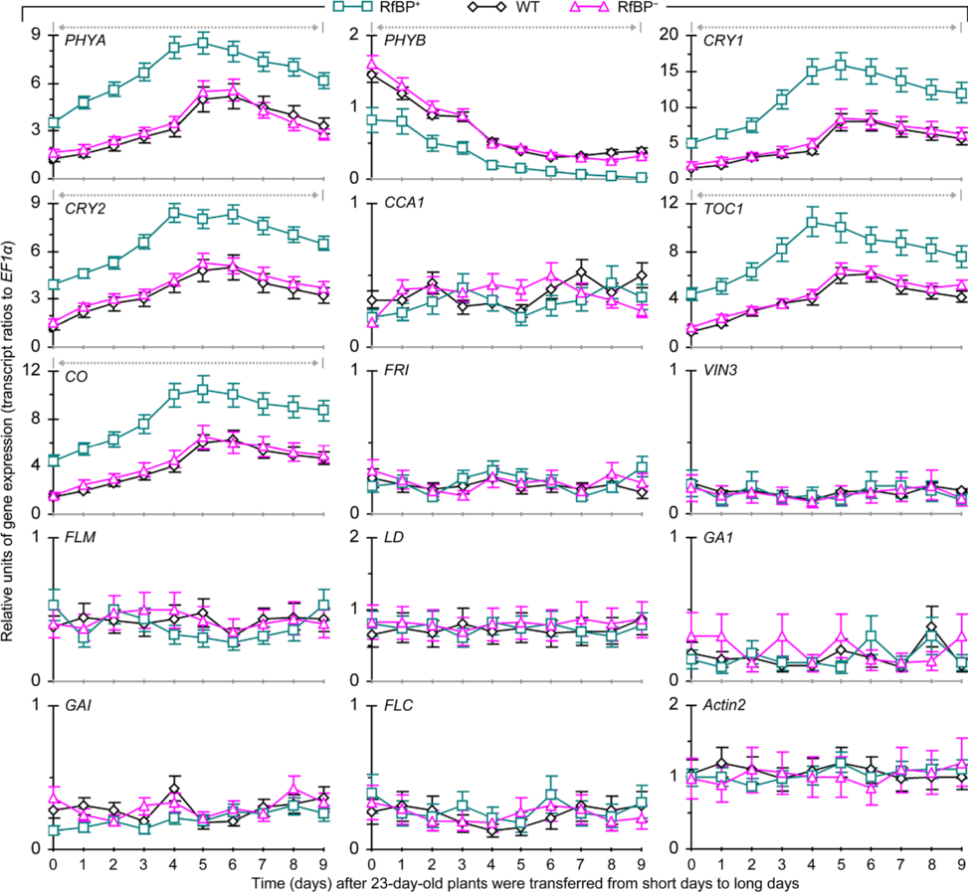
**The expression of flowering regulatory genes in leaves of WT, RfBP** ^**+**^ **, and RfBP** ^**−**^ **plants grown under inductive photoperiod.** Gene expression in the two youngest leaves was analyzed by real-time RT-PCR at the indicated times. Data shown in curves are mean values ± standard deviation bars of results from three independent experiments each containing three repeats and five plants per repeat. Gray dashed and bidirectional arrowheads indicate significant differences between RfBP^+^ and WT or RfBP^−^ at the range of time intervals based on analysis of variance and least significant difference test (*P* < 0.01).

As shown in Figure 7, inductive photoperiod caused different effects on the foliar expression of genes assigned to different floral pathways and the effects were also different in RfBP^+^ from WT and RfBP^−^. In all plants, inductive photoperiod did not cause evident effect on *CCA1* or genes assigned to vernalization, GA, and autonomous pathways in comparison with transcript ratios between reference genes *Actin2* and *EF1α*. Inductive photoperiod repressed the expression of *PHYB* and repression extents were significantly (*P* < 0.01) lower in RfBP^+^ leaves than in leaves of WT or RfBP^−^. In comparison to transcript ratios between *Actin2* and *EF1α*, expression levels of floral promoting photoperiod genes *PHYA*, *CRY1*, *CRY2*, *TOC1*, and *CO* in leaves of all plants were increased by inductive photoperiod. These genes were expressed in a similar chronological pattern. Expression levels were increased slightly in 3 days in RfBP^+^ and 5 days in WT and RfBP^−^, reached the highest levels in the next two days, and then declined in all plants. At every time point, extents by which inductive photoperiod acted to enhance the expression of *PHYA*, *CRY1*, *CRY2*, *TOC1*, and *CO* were significantly (*P* < 0.01) greater in RfBP^+^ leaves than in leaves of WT or RfBP^−^.

**Figure 8:**
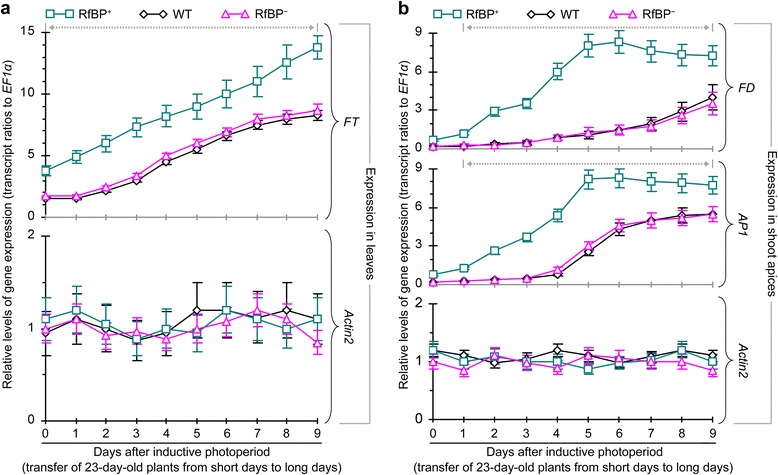
**The expression of** ***FT*** **,** ***FD*** **, and** ***AP1*** **under inductive photoperiod.** Gene expression in the two youngest leaves **(a)** and shoot apices **(b)** was analyzed by real-time RT-PCR at the indicated times. Data shown in curves are mean values ± standard deviation bars of results from three independent experiments each containing three repeats and five plants per repeat. Gray dashed and bidirectional arrowheads indicate significant differences between RfBP^+^ and WT or RfBP^−^ at the range of time intervals based on analysis of variance and least significant difference test (*P* < 0.01).

In all plants, inductive photoperiod caused enhancements in the foliar expression of *FT* (Figure 8a) and the expression of *FD* and *AP1* in shoot apices (Figure 8b). Nevertheless, enhancement extents were significantly (*P* < 0.01) greater in RfBP^+^ than in WT or RfBP^−^. In all plants, moreover, expression levels of *FT* in leaves and expression levels of *FD* and *AP1* in shoot apices were increased in six days and then the foliar expression of *FT* was continuously increased (Figure 8a) but the apical expression of *FD* and *AP1* remained stable till the ninth day (Figure 8b).

Taken together, these analyses suggest that the de novo expression of RfBP in Arabidopsis enhances the expression of *FT* and floral promoting photoperiod genes in leaves and also enhances the expression of *FD* and *AP1* in the shoot apex under inductive photoperiod. Gene expression enhancements are significant in the RfBP^+^ plant compared to WT or RfBP^−^ background.

### Reduction of leaf flavin content is responsible for enhancements of the gene expression under inductive photoperiod

**Figure 9:**
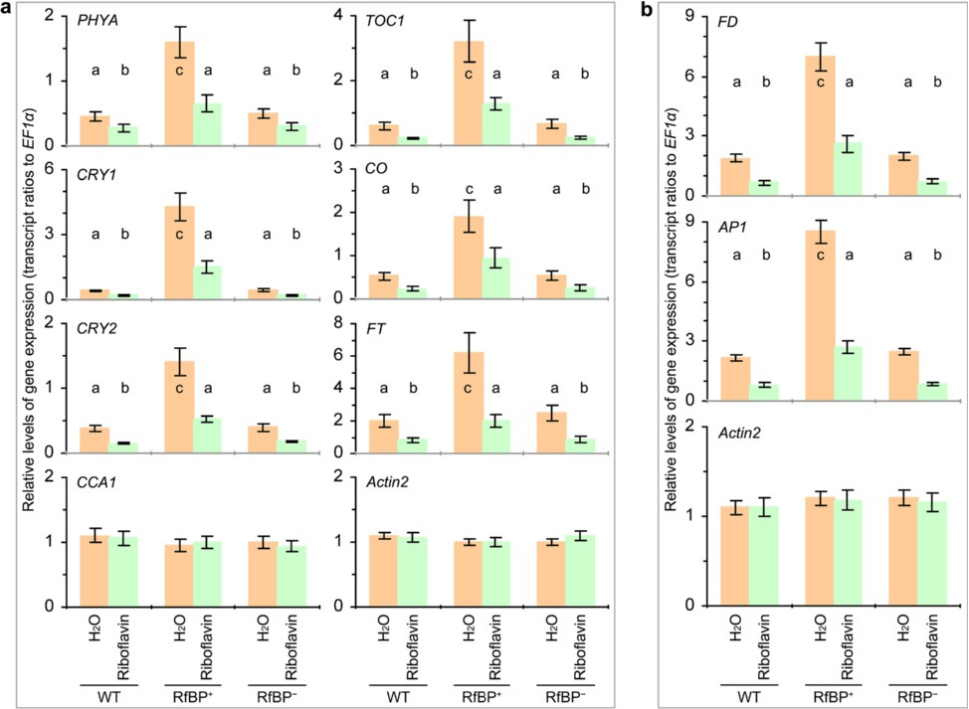
**The effects of riboflavin feeding on the expression of flowering regulatory genes under inductive photoperiod.** Plants were grown in short days for 23 days and transferred to long days. Immediately after plant transfer, H_2_O or an aqueous riboflavin solution was applied by spraying over plant tops. Four days later, the expression of *FT* and photoperiod genes in leaves **(a)** and the expression of *FD* and *AP1* in shoot apices **(b)** were analyzed by real-time RT-PCR using *EF1α* and *Actin2* as reference genes. Data shown are mean values ± standard deviation bars of results from three independent experiments each containing three repeats and 15 plants per repeat. Different letters in bar graphs indicate significant differences by analysis of variance and least significant difference test (*P* < 0.01).

To correlate leaf flavin content with RfBP-enhanced gene expression under inductive photoperiod, we tried to increase flavin levels by feeding plants with riboflavin and analyzed *PHYA*, *CRY1*, *CRY2*, *CCA1*, *TOC1*, *CO*, *FT*, *FD*, and *AP1* expression at the fifth day after inductive photoperiod, a time point at which these genes are highly expressed in leaves or shoot apices in the absence of riboflavin feeding (Figures 7 and 8). Under inductive photoperiod, feeding plants with riboflavin caused substantial increases in leaf concentrations of all flavins, and flavin levels in riboflavin-fed RfBP^+^ were retrieved to the approximations in water-treated WT plants (Additional file 3: Figure S3). RfBP^−^ resembled WT in the riboflavin-feeding effects on leaf flavin content (Additional file 3: Figure S3). In all plants, *CCA1* expression in leaves was unaffected, but the foliar expression of *PHYA*, *CRY1*, *CRY2*, *TOC1*, *CO*, and *FT* in leaves (Figure 9a) and the expression of *FD* and *AP1* in shoot apices (Figure 9b) were decreased by the riboflavin feeding treatment compared to water. Riboflavin-fed RfBP^+^ plants performed similarly to water-treated WT or RfBP^−^ plants in gene expression. In RfBP^+^, therefore, enhancements of *FT* and photoperiod gene expression in leaves, and enhancements of *FD* and *AP1* expression in the shoot apex, are caused by the reduction of leaf flavin concentrations.

## Discussion

The well-demonstrated developmental role of oviparous RfBP in riboflavin binding and redistribution [13,28,32] inspired the idea to manipulate plant riboflavin content by engineering with the turtle RfBP [11]. Its activity in riboflavin binding allows for the function in modulating free flavin concentrations in transgenic plants [11]. On this basis, in the present study we have characterized the serendipitous role of the RfBP protein in affecting flowering time after de novo expression in Arabidopsis. We investigated Arabidopsis RfBP^+^ and RfBP^−^ lines in comparison with the WT plant (Figure 1) and demonstrated that RfBP-caused downregulation of free flavin content in leaves (Figure 1) induced the early flowering phenotype (Figure 2). By feeding plants with riboflavin to increase the intrinsic content of free flavins and determining the subsequent effect on flowering time, analyzing the pharmacological data together with those about the RfBP^+^ vs. RfBP^−^ effects, we were able to attribute the early flowering phenotype to the reduction of free flavin concentrations in leaves (Figure 3) on the basis of RfBP binding with riboflavin inside leaf cells [11].

Riboflavin is a venerable multifaceted player in tremendous biochemical processes and frequently receives renascent attentions with newly discovered functions [1–11]. Since its discovery in 1879 and biochemical characterization in 1933, a variety of physiological roles that flavins play in plants have been extensively studied [3,53]. In particular, previously unappreciated functions have been often reported in recent 10 years. For example, genetic modification of the riboflavin biosynthesis pathway alters some aspects of plant development, such as leaf senescence regulated by the COS1 protein, an essential component of the jasmonic acid signaling pathway [54]. In fact, COS1 is the lumazine synthase [54], which catalyzes the penultimate step of the riboflavin biosynthesis pathway [55]. Arabidopsis mutants that have partial defect in *COS1* and partial decrease in riboflavin content compromise the regulatory role of jasmonic acid in leaf senescence [54]. In plants, moreover, externally applied riboflavin induces resistance to pathogens by priming of defense responses in a manner of salicylic acid dependence or independence according to the type of pathogens, biotrophic or necrotrophic [6,10]. Externally applied riboflavin also induces plant growth enhancement by activating the ethylene signaling pathway [56]. These findings suggest that changes in riboflavin content cause physiological and pathological responses by affecting phytohormone signaling pathways. Through studies detailed here, novel functions of flavins have been extended from cellular signaling to flowering time control in relation to the expression of photoperiod and flowering time genes.

The expression of photoperiod and flowering time genes is implicated in RfBP-induced flowering based on several lines of evidence (Figures 4, 5, 6, 7, 8 and 9). First, RfBP^+^ causes enhanced expression of five photoperiod genes (*PHYA*, *CRY1*, *CRY2*, *TOC1*, and *CO*), which are flowering activators [34,35,40], in leaves under long day (Figure 4) or inductive photoperiod (Figure 7) conditions. By contrast, PHYB is a flowering repressor [44,57] and *PHYB* expression is repressed by RfBP in contrast to the early flowering phenotype and RfBP-enhanced expression of the floral promoting photoperiod genes (Figures 4 and 7). In addition, *CCA1* is highly expressed in the early phase and its expression declines in the late phase of day (46,49), explaining why RfBP is unable to affect *CCA1* expression. Similarly, genes assigned to autonomous, gibberellin, and vernalization pathways are not related to RfBP-induced early flowering (Figure 4). Second, enhanced expression of the photoperiod genes was correlated with enhanced expression of *FT* in leaves (Figures 5 and 8). The FT protein is the florigen that can moves from leaves to shoot apices [51,52], where it functions with FD to activate AP1 for the growth of floral organs [42,43]. Third, concomitantly enhanced expression of *FT* and photoperiod genes was further correlated with the synchronized expression of *FD* and *AP1* in the shoot apex (Figures 6, 8, and 9), while synchronized expression of *FD* and *AP1* in the shoot apex initiates floral organ formation [42,43,58,59]. The role of RfBP in gene expression is attributable to reduction of free flavin levels in leaves (Figure 9).

As flavins anticipate in numerous biochemical processes, it is difficult to elucidate the functional relationship between downregulated flavin concentrations and the photoperiod pathway. A possible mediator is H_2_O_2_, a cellular signal that can be induced by the de novo RfBP expression and downregulation of free flavin content inside Arabidopsis leaves [11]. H_2_O_2_ has been implicated in crosstalk with flowering regulators [60] and actually participates in the regulation of flowering time [61–64]. For example, flowering is promoted when cytosolic H_2_O_2_ levels are elevated by the activity of chloroplastic lipoxygenase or ascorbate peroxidase in Arabidopsis [61,62]. As downregulation of free flavin concentrations in leaves by RfBP induces the production of the H_2_O_2_ signal and its translocation from the apoplast to the cytosol [11], the signal may act in turn to promote flowering [61,62]. Alternatively, H_2_O_2_ may be generated through electron leakage from the mitochondrial electron transport chain due to shortage of FMN and FAD, which serve as redox centers in the chain [65–68].

## Conclusions

Meticulous phenotypic observations indicate that early flowering is a constant character conferred by the de novo expression of RfBP in transgenic Arabidopsis plants grown in short days and long days. The phenotype is caused indirectly by downregulation of free flavin concentrations in leaves based on pertinent analyses of the RfBP^+^ vs. RfBP^−^ effects, as well as the pharmacological consequence from the riboflavin feeding treatment, performed under long days and inductive photoperiod. Under both conditions, reduction of leaf flavin content induces the expression of floral promoting photoperiod genes in leaves, coincident expression of the florigen gene *FT* in leaves, and synchronized expression of the flowering regulatory gene *FD* and the floral meristem identity gene *AP1* in the shoot apex. We don’t have evidence to show the connection between changes in flavin concentrations and any of the floral regulators. In fact, we found the early flowering phenomenon by accident, but we don’t know what it means with respect to photoperiod gene expression and flowering time control.

## Methods

### Plant growth conditions and flowering observations

Plants were grown in pots containing potting soil [69] under the environment-controlled conditions: 22 ± 1°C, 55% humidity, short days or long days, and light at 200 μM quanta/m^2^/s. The flowering phenotype was characterized by two criteria: days to flowering and rosette leaf number [70].

### Gene expression analyses

Total RNA was isolated from the two youngest expanded leaves or shoot apices and subjected to real-time RT-PCR or Northern (RNA) blotting analyses using the constitutively expressed *EF1α* and/or *Actin2* genes as references. Real-time RT-PCR was performed with specific primers (Additional file 4: Table S1) as previously described [71,72]. The expression level of a tested gene was quantified as the ratio between transcript amounts of the gene and *EF1α*. Northern blots were hybridized to the *RfBP*-specific probe labeled with digoxigenin (Novagen, EMD Biosci., Inc., WI, USA).

### Protein analyses

A histidine (His) tag had been added to the C-terminus of RfBP in the transformation construction and was used to facilitate purification of plant protein preparations by nickel chromatography [11]. The two youngest expanded leaves were excised and used in isolation of total proteins from 10 mg fresh leaves as previously described [73]. Isolated proteins were bound to nickel-polystyrene beads according to the manufacturer’s instruction (Amersham Biosciences Corp., Piscataway, NJ, USA), eluted with aqueous solutions of imidazole at 100, 150, and 300 mM, respectively. The 200-mM imidazole eluent was treated with the Novagen Enterokinase Cleavage Capture Kit (EMD Biosciences Inc., Darmstadt, Germany) to remove the His tag and analyzed by tricine sodium dodecyl sulfate polyacrylamide gel electrophoresis [71]. Proteins were visualized by gel staining with Coomassie G-250.

### Flavin measurements

All operations were in subdued light. Riboflavin, FMN, and FAD were extracted using a previously described method [11,74]. Leaf samples (1 g/treatment) were ground on the ice with 2 ml cold extraction buffer A (pH6.9) containing 5 mM NaH_2_PO_4_^.^2H_2_O, 5 mM Na_2_HPO_4_^.^12H_2_O, 0.2 M NaCl, 0.5 mM phenylmethylsulfonyl fluoride, and 1 mM ethylene dianetetra-acetic acid. Homogenate was centrifugated at 4°C and 12,000 g for 10 minutes. Supernatant was divided into two groups. In the first group, 200 μl supernatant was supplemented with 1 ml buffer B made of 10% trichloroacetic acid in 0.1 M ammonium acetate (pH6.1). The mixture was centrifuged at room temperature (12,000 g, 10 minutes) and the new supernatant was regarded as a preparation of total flavins [74]. In the second group, 500 μl supernatant was loaded into a Microcon YM-3 (3 kDa NMWL) ultrafiltration spin column (Millipore, Billerica, MA, USA). The column was spun at 4°C and 14,000 g for 15 minutes. Filtrate of 200 μl was shifted into an Eppendorf tube, supplemented with 1 ml buffer B. The mixture was centrifuged at room temperature (12,000 g, 10 minutes) and the final supernatant was regarded as a preparation of free flavins. The preparations of total and free flavins were filtrated separately with 0.22 μm blend cellulose ester filters. Each filtrate of 20 μl was analyzed by high performance liquid chromatography [75] with the Agilent 1200 HPLC system (Agilent Tech. Inc., Santa Clara, CA, USA). Concentrations of riboflavin, FMN, and FAD in the preparations were determined by reference to similar analysis of the inner standards [75] and quantified in contrast to plant weight.

### Riboflavin feeding experiments

The riboflavin (EMD Biosci., Inc., Darmstadt, Germany) feeding experiments were performed on plants grown under long day and inductive photoperiod conditions, respectively. Plants were treated by spraying over tops with an aqueous solution of 0.2 mM riboflavin, made in ultrapure water produced by the EliX10/Milli-Q Synthesis A10 ultrapure water system (Merck Millipore Corporation, Billerica, MA, USA), and treated similarly with ultrapure water in the experimental control group. Flavin measurements and gene expression analyses were performed on the two youngest expanded leaves. Flowering time and the rosette leaf number were monitored.

### Data treatment

All experiments were carried out at least three times with similar results. Quantitative data were analyzed with the IBM SPSS19.0 software package (IBM Corporation, Armonk, NY, USA; http://www-01.ibm.com/software/analytics/spss/) according to instructions in a text book that describes in details analysis methods using IBM SPSS19.0 [76]. Homogeneity-of-variance in data was determined by Levene test, and formal distribution pattern of the data was confirmed by Kolmogorov-Smirnov test and P-P Plots [76]. Then, data were analyzed by analysis of variance and least significant difference test [77].

## Additional files

Additional file 1: Figure S1. Flowering characteristics of different RfBP-expressing Arabidopsis lines in comparison with the WT plant in long days. Additional file 2: Figure S2. The effects of riboflavin feeding treatment on expression of photoperiod genes in long days. Additional file 3: Figure S3. The effects of riboflavin feeding treatment on flavin concentrations in leaves under inductive photoperiod. Additional file 4: Table S1. Information on genes tested and primers used in this study.

## Abbreviations

AP1: APETALA1
CCA1: CIRCADIAN CLOCK-ASSOCIATED1
CO: CONSTANS
CRY: Cryptochrome
FAD: Flavin adenine dinucleotide
FLC: FLOWERING LOCUS C
FMN: Flavin mononucleotide
FLM: FLOWERING LOCUS M
FRY: FRIGIDA
FT: FLOWERING LOCUS T
GA: Gibberellin
GAI: GA INSENSITIVE
GA1: GA REQUIRING 1
LD: LUMINIDEPENDENS
PHY: Phytochrome
RfBP: riboflavin-binding protein
RfBP^+^: RfBP-expressing transgenic Arabidopsis line
RfBP^−^: RfBP-silenced Arabidopsis line generated under RfBP^+^ background
SOC1: Suppressor of overexpression of CO1
TOC1: Timing of cab expression1
VIN3: Vernalization insensitive 3
