# Supplementary material for: Downregulation of leaf flavin content induces early flowering and photoperiod gene expression in Arabidopsis
Source: BMC Plant Biol. 2014 Sep 9;14:237. doi: 10.1186/s12870-014-0237-z (PMC4172855; doi:10.1186/s12870-014-0237-z)
Supplement: Additional file 1: Figure S1. — Flowering characteristics of different RfBP-expressing Arabidopsis lines in comparison with the WT plant in long days. [file 12870_2014_237_MOESM1_ESM.doc]

**Additional file**

**
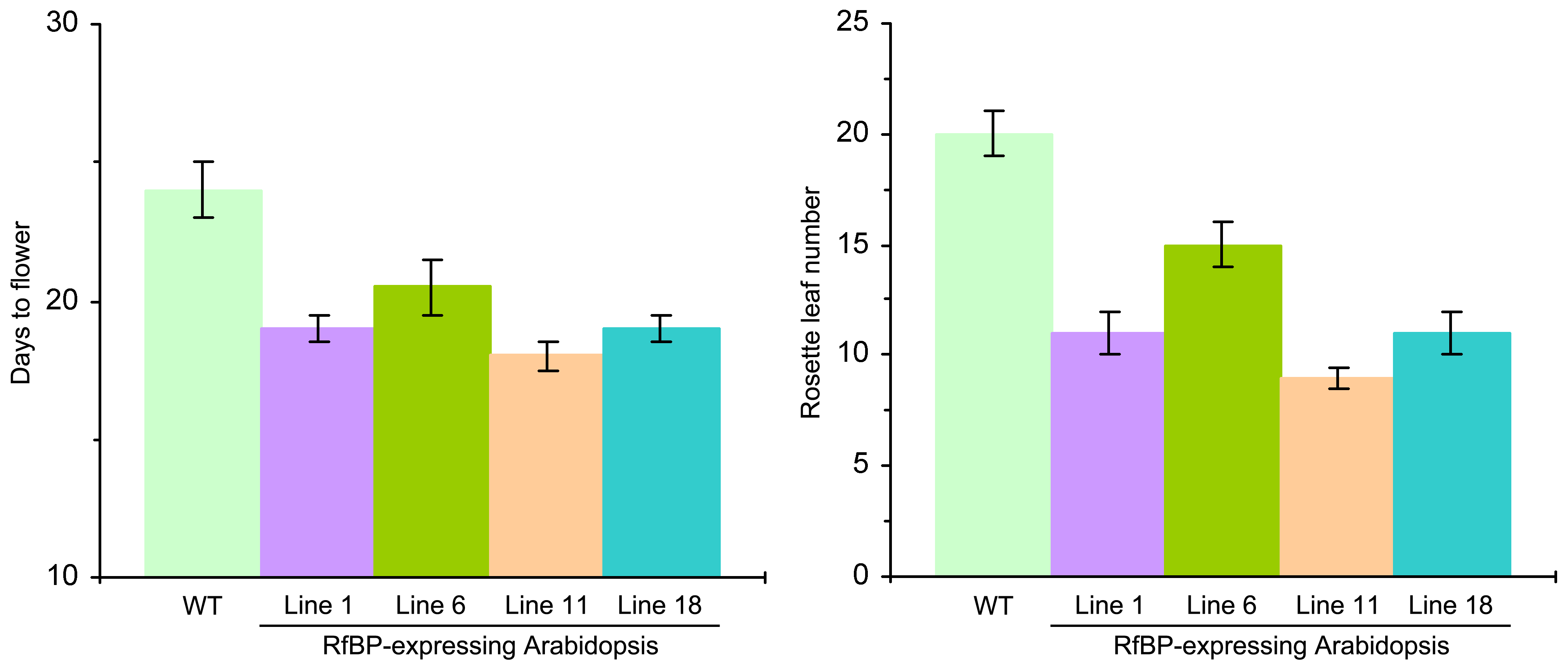
Additional file 1: Figure S1.**Flowering characteristics of different RfBP-expressing Arabidopsis lines in comparison with the WT plant in long days. Line 11 was further investigated and renamed RfBP^+^ in this study. Data shown are mean values ± standard deviation bars of results from three independent experiments each containing three repeats and 15 plants per repeat.
