# Supplementary material for: Downregulation of leaf flavin content induces early flowering and photoperiod gene expression in Arabidopsis
Source: BMC Plant Biol. 2014 Sep 9;14:237. doi: 10.1186/s12870-014-0237-z (PMC4172855; doi:10.1186/s12870-014-0237-z)
Supplement: Additional file 2: Figure S2. — The effects of riboflavin feeding treatment on expression of photoperiod genes in long days. [file 12870_2014_237_MOESM2_ESM.doc]

**Additional file**

**
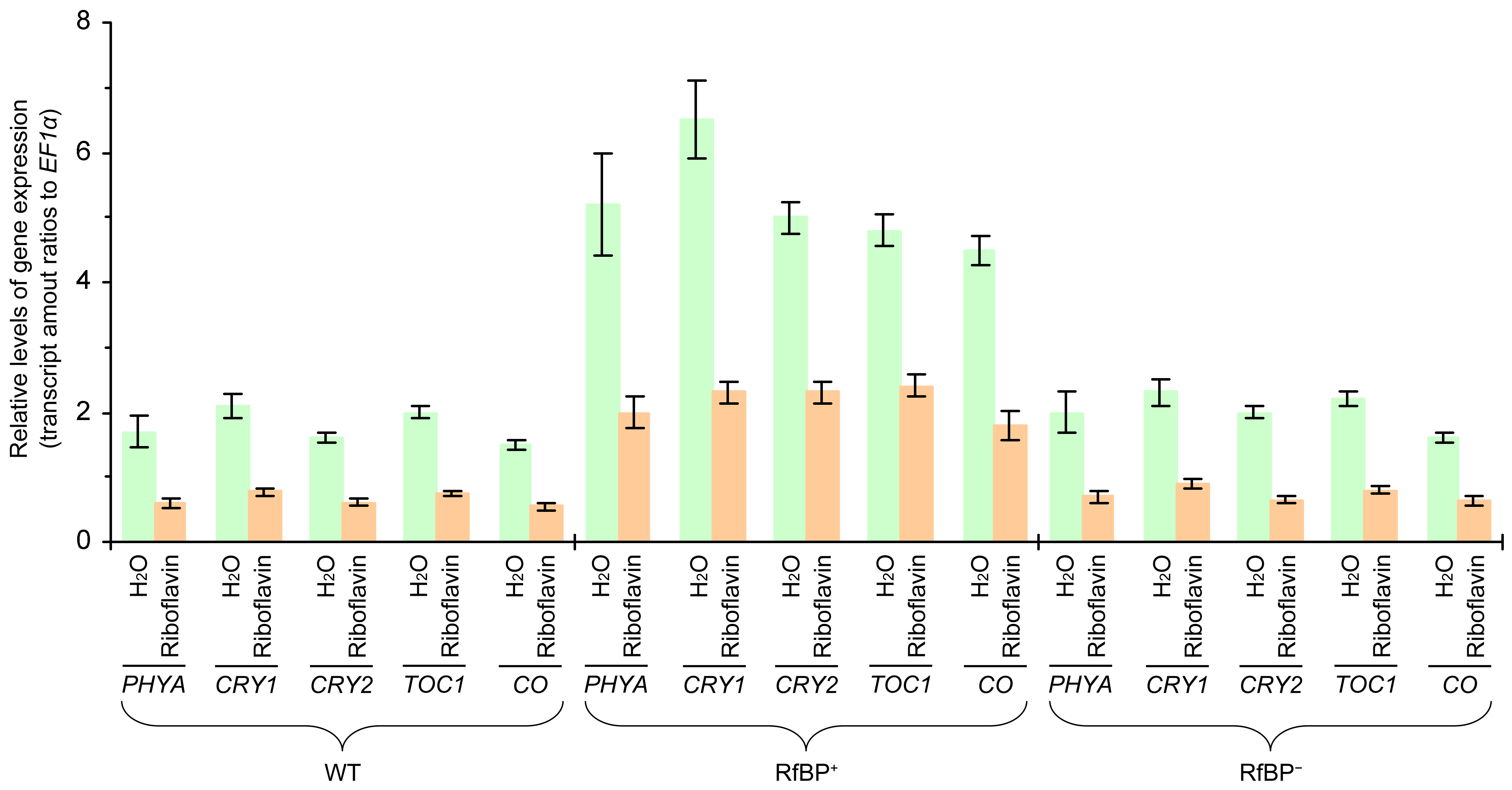
**

**Additional file 2: Figure S2.** The effects of riboflavin feeding treatment on expression of photoperiod genes in long days. Ten-day-old plants were fed with riboflavin or water. Gene expression in leaves was analyzed by real-time RT-PCR using the constitutively expressed *EF1α* gene as a reference. Data shown are mean values ± standard deviation bars of results from three independent experiments each containing three repeats and 15 plants per repeat.
