# Supplementary material for: Downregulation of leaf flavin content induces early flowering and photoperiod gene expression in Arabidopsis
Source: BMC Plant Biol. 2014 Sep 9;14:237. doi: 10.1186/s12870-014-0237-z (PMC4172855; doi:10.1186/s12870-014-0237-z)
Supplement: Additional file 3: Figure S3. — The effects of riboflavin feeding treatment on flavin concentrations in leaves under inductive photoperiod. [file 12870_2014_237_MOESM3_ESM.doc]

**Additional file**

**
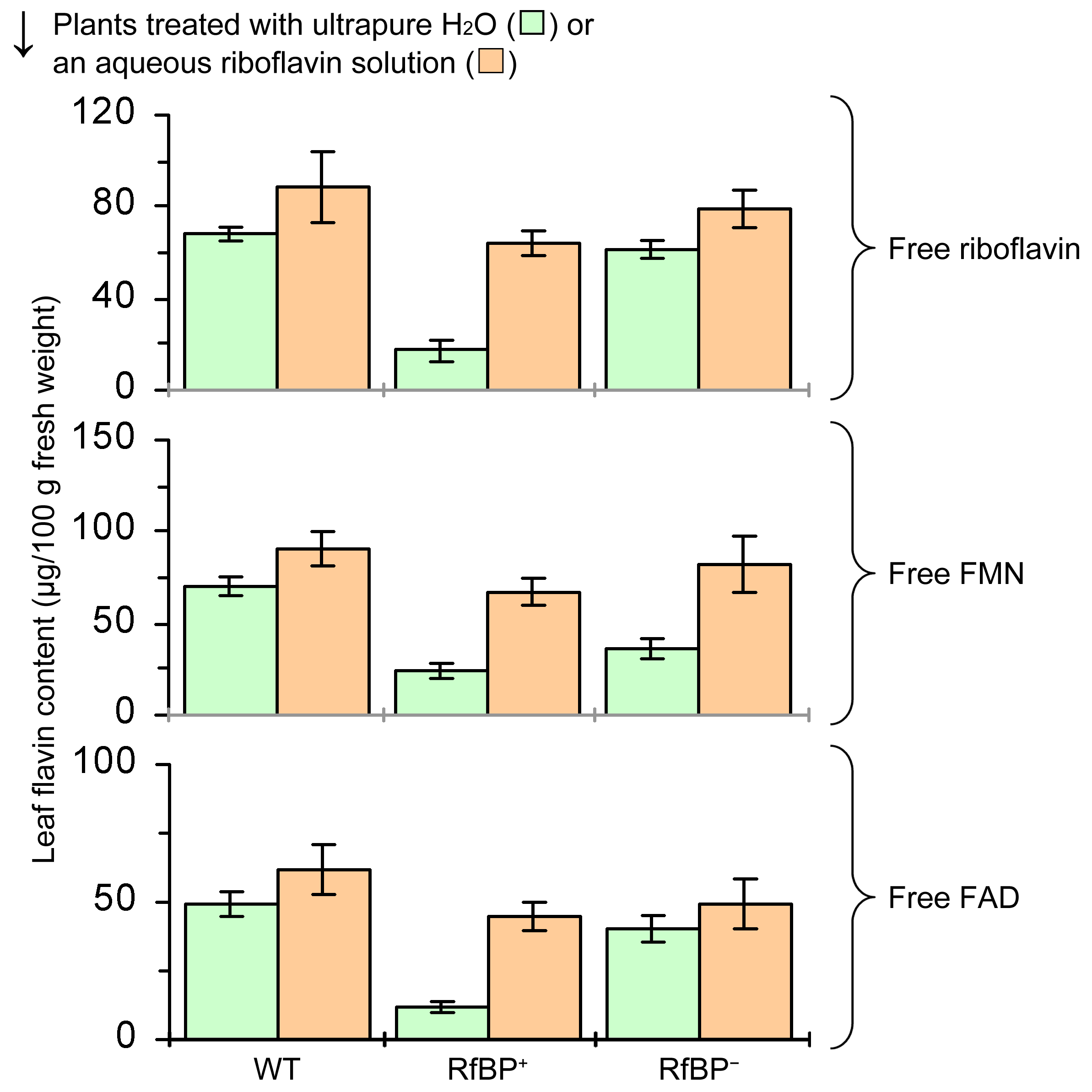
**

**Additional file 3: Figure S3.** The effects of riboflavin feeding treatment on flavin concentrations in leaves under inductive photoperiod. Plants were grown in short days for 23 days and transferred to long days. Immediately after plant transfer, H_2_O or an aqueous riboflavin solution was applied by spraying over plant tops. Two days later, concentrations of free flavins in leaves were determined. Data shown are mean values ± standard deviation bars of results from three independent experiments each containing three repeats and 15 plants per repeat.
