# Supplementary material for: Downregulation of leaf flavin content induces early flowering and photoperiod gene expression in Arabidopsis
Source: BMC Plant Biol. 2014 Sep 9;14:237. doi: 10.1186/s12870-014-0237-z (PMC4172855; doi:10.1186/s12870-014-0237-z)
Supplement: Additional file 4: Table S1. — Information on genes tested and primers used in this study. [file 12870_2014_237_MOESM4_ESM.doc]

**Additional file**

**Additional file 4: Table S1.** Genes tested and primers used in this study

| Gene* | Primers / product size (bp) | Use for |
| --- | --- | --- |
| *RfBP* | 5’-GGCTGACAGATTGGGAATGGGATG-3’  5’-TTTCGGAAGTTAAGTACCTGATGG-3’/ 201 | Real-time RT-PCR |
| *EF1α* | 5’-AGACCACCAAGTACTACTGCAC-3’, 5’-CCACCAATCTTGTACACATCC-3’ / 496 | RT-PCR |
| *Actin2* | 5’-CCCCTGAGGAGCACCCAGTTCTA-3’, 5’-CATACCCCTCGTAGATTGGCACAG-3’ / 219 | RT-PCR and real-time RT-PCR |
| *PHYA* | 5’-GTATGTGGAGTGTCTGTTGTGTGT-3’  5’-CTTCATCATTACTTGACTTGTGGA -3’/ 412 | RT-PCR |
|  | 5’-TCCTGCCACCGACATCCCTCA-3’  5’-GCATCCCCTTCTCCATCTTCC-3’/ 249 | Real-time RT-PCR |
| *PHYB* | 5’-TTACAGGCTCTTCCTGGTGGAGAT-3’  5’-GGTATGCAGCGAGAAGAAGTGTGA-3’/ 500 | RT-PCR |
|  | 5’-TGTCCAGGTGAAGGTCTGCC-3’, 5’-ACAATGGTCGCTTTCGAGGTA-3’ / 202 | Real-time RT-PCR |
| *CRY1* | 5’-CCAGACTCAACCCAAACAGGAGAT-3’  5’-CACCGGAGTTACAGCCCTTATGAT-3’/ 500 | RT-PCR |
|  | 5’-GCGGATTTAGAAAGCGATGC-3’, 5’-AGGTGCGTTCCACGGATG-3’ / 195 | Real-time RT-PCR |
| *CRY2* | 5’-CGCCGAGAGATATCCACTCTGATT-3’  5’-ACGTTCCACCAGCTTCTCCTTTAC-3’/ 504 | RT-PCR |
|  | 5’-TTCGTTAGTTCGGGACCATACC-3’, 5’-CACGCCCAAATCGCTTCA-3’ / 240 | Real-time RT-PCR |
| *CCA1* | 5’-AGAGCCAGATAGTCATCCCCACAC-3’  5’-CTGAACCCTTTGCATCACTCTCAG-3’/ 491 | RT-PCR |
|  | 5’-AGCAACGTGAAAGGTGGACTG-3’, 5’-GCCGTGGAGGAGCAATAGC-3’ / 223 | Real-time RT-PCR |
| *TOC1* | 5’-CAAGACGAAGTCCCTGTCGTTGTA-3’  5’-AGCAGAGGACTCTCCGATCTTCAA-3’/ 501 | RT-PCR |
|  | 5’-GGGTTTGGCGGAGAAGTT-3’, 5’-CGCTCAAGGGAATGTGGATA-3’ / 294 | Real-time RT-PCR |
| *CO* | 5’-GCCTACTTGTGCATGAGCTGTGAT-3’ 5’-GTCTGTGGTACGCTGCAGTTTTGT-3’/ 509 | RT-PCR |
|  | 5’-GTGATAAGGATGCCAAGGAG-3’ 5’-AGTTTAAGCGGAACAACTCTA-3’ / 223 | Real-time RT-PCR |
| *FRI* | 5’-CGAATAAACCGGAGGGGGAACGTA-3’  5’-TGAAGGAACACCAAAACAAGCAAC-3’/ 434 | RT-PCR |
|  | 5’-ACATATACGCGAATATCTCTG-3’  5’-TCTTCACCTTCCCTTTACCAC-3’/ 231 | Real-time RT-PCR |
| *VIN3* | 5’-GCGTATTGCGGTAAAGATAATGAC-3’  5’-TTTTGTGTAGAAGAAGACGGCTCC-3’/ 428 | RT-PCR |
|  | 5’-TCTGTCTCAGAGTGGTTTCC-3’  5’-CAGTGTTCAGTGTTGTCCTT-3’/ 226 | Real-time RT-PCR |
| *FLM* | 5’-CTCCGTCGCTGTTGTCGTCGTATC-3’  5’-TTCCTCTCTCATCATCTGTTGCCA-3’/ 410 | RT-PCR |
|  | 5’-ATCGTTATGAAATACAACAT-3’  5’-CCTAGCTCTACTTACGGACA-3’/ 200 | Real-time RT-PCR |
| *LD* | 5’-GGAGAGTAGCCGCTGGTGGTAATA-3’ 5’-AGTTGATGGAGTGGGAGATGGAAG-3’ / 482 | RT-PCR |
|  | 5’-CGAGTTATTAGCTGCGTTAC-3’ 5’-ATTAGTTGATGGAGTGGGAG-3’ / 202 | Real-time RT-PCR |
| *GA1* | 5’-TCTCTCCTTCCTCTACCGCTTTTG -3’  5’-GTTACTGCTTGGTTTGATTGCCCC-3’/ 400 | RT-PCR |
|  | 5’-TTCTTCTTTCCTTACCATCTC-3’  5’-TGCATTACTATTACTTCCAAC-3’/ 205 | Real-time RT-PCR |
| *GAI* | 5’-TGCGATCGATAAGGTTCTTGGTGT-3’  5’-AGGTGGCTATGAGCGGTCGTGTGT-3’/ 440 | RT-PCR |
|  | 5’-TCCACCGGCACCGGATAATTT-3’  5’-TCGCACCAGGTCGTCCCAAGA-3’/ 221 | Real-time RT-PCR |
| *FLC* | 5’-AGCTCGTCAGCTTTCTGTTCTCTG-3’  5’-AGTAGTGGGAGAGTCACCGGAAGA-3’ / 492 | RT-PCR |
|  | 5’-GTCGCTCTTCTCGTCGTCTC-3’ 5’-TCTCAAGGTGTTCCTCCAGT-3’ / 250 | Real-time RT-PCR |
| *FT* | 5’-TACGAAAATCCAAGTCCCACTG-3’, 5’-AAACTCGCGAGTGTTGAAGTTC-3’ / 219 | RT-PCR and real-time RT-PCR |
| *AP1* | 5’-GCACATCCGCACTAGAAAAAA-3’, 5’-CTTCTTGATACAGACCACCCA-3’ / 275 | RT-PCR and real-time RT-PCR |
| *FD* | 5’-TAATCTTCATACCCACCATCAC-3’, 5’-CAATCCCCAAAAGAGAAACAAG-3’ / 377 | RT-PCR |
|  | 5’-TCCACCATTGTCACTGCTCT-3’ 5’-TACGCTTATACCTTCTGTCTCC-3’ / 220 | Real-time RT-PCR |
